# Identification of α1-Antitrypsin as a Potential Candidate for Internal Control for Human Synovial Fluid in Western Blot

**DOI:** 10.4172/2161-1149.S6-006

**Published:** 2015-05-28

**Authors:** Shaowei Wang, Jingming Zhou, Xiaochun Wei, Pengcui Li, Kai Li, Dongming Wang, Fangyuan Wei, Jianzhong Zhang, Lei Wei

**Affiliations:** 1 Department of Orthopedics, the Second Hospital, Shanxi Medical University, Taiyuan, Shanxi, China; 2 Department of Orthopedics, Warren Alpert Medical School of Brown University/Rhode Island Hospital, Providence RI, USA; 3 Department of Orthopedics, Sir Run Run Shaw Hospital, Medical School of Zhejiang University, Hangzhou, Zhejiang, China; 4 Foot and Ankle Orthopaedic Surgery Center, Beijing Tongren Hospital, Capital Medical University, Beijing, China

**Keywords:** α1-Antitrypsin, Loading control, Synovial fluid, Western blot

## Abstract

Western blot of synovial fluid has been widely used for osteoarthritis (OA) research and diagnosis, but there is no ideal loading control for this purpose. Although β-actin is extensively used as loading control in western blot, it is not suitable for synovial fluid because it is not required in synovial fluid as a cytoskeletal protein. A good loading control for synovial fluid in OA studies should have unchanged content in synovial fluids from normal and OA groups, because synovial fluid protein content can vary with changes in synovial vascular permeability with OA onset. In this study, we explore the potential of using α1-antitripsin (A1AT) as loading control for OA synovial fluid in western blot. A1AT level is elevated in inflammatory conditions such as rheumatoid arthritis (RA). Unlike RA, OA is a non-inflammation disease, which does not induce A1AT. In this study, we identified A1AT as an abundant component of synovial fluid by Mass Spectrometry and confirmed that the level of A1AT is relative constant between human OA and normal synovial fluid by western blot and ELISA. Hence, we proposed that A1AT may be a good loading control for western blot in human OA synovial fluid studies provided that pathological conditions such as RA or A1AT deficiency associated liver or lung diseases are excluded.

## Introduction

Synovial fluid has been widely used for research, diagnosis, and treatment of joint diseases, such as osteoarthritis (OA). Although β-actin is extensively used as loading control in western blot [1], it is not an established control for synovial fluid. A good loading control for synovial fluid in OA studies should have unchanged content in synovial fluids from normal and OA groups, because synovial fluid protein content can vary with changes in synovial vascular permeability with OA onset. In this study, we are the first lab to explore the potential of using α1-antitripsin (A1AT) as loading control for synovial fluid in western blot.

A1AT, a 52-kDa protease inhibitor, is synthesized in the endoplasmic reticulum of the liver cells, released to blood, and diffused to lung epithelial cells [2]. In lungs, A1AT balances the activity of neutrophil elastase [3], which is released by neutrophils to digest damaged cells and bacteria in response to inflammation and infection [4]. A1AT also blocks apoptosis in lung endothelial cells by inhibiting caspase-3 activity [3]. A1AT deficiency can lead to lung damage by overactivated neutrophil elastase and caspase-3 [5]. As an acute phase reactant, A1AT is elevated in acute and chronic inflammatory conditions, infections, and with some cancers [6]. In synovial fluid, A1AT also plays a proteinase inhibitory role [7]. A1AT level in synovial fluid is lower but highly correlated with that in serum [8]. In rheumatoid arthritis (RA), A1AT level in synovial fluid is significantly elevated compared to normal synovial fluid [9], which is consistent with the fact that RA involves chronic, systemic inflammation, and the presence of neutrophils in RA synovial fluid [10].

In this study, we identified and confirmed that A1AT is abundant and relatively constant in OA and normal synovial fluid by Mass Spectrometry, western blot and ELISA respectively. Since there is no established loading control for western blot with human synovial fluid samples, we proposed that A1AT may be a good candidate for internal control in human synovial fluid studies.

## Materials and Methods

The study was approved by the Institutional Review Board at Rhode Island Hospital of the US and Shanxi medical University of China, and informed consent was obtained from each donor.

### Enrollment of patients

OA synovial fluid was obtained during patient OA knee joint replacement (N=19, 8 male, 11 female, mean ± SD age 65.5 ± 10.3, range 52–86). Normal control synovial fluid was obtained from healthy volunteers and the contralateral uninjured knee of patients undergoing unilateral ACL reconstruction (ACLR) (N=20, 13 male, 7 female, mean ± SD age 29.3 ± 10.9, range 14–52).

Patients who had inflammatory joint disease, acute major trauma, malignant tumors, or abnormal renal and liver function were excluded from the study. Patients who took corticosteroid treatment within the 3 months preceding surgery were also excluded from the study.

### Collection and storage of synovial fluid

A volume of 0.5–5 ml of synovial fluid was aspirated from the knee joint just before total knee replacement or arthroscopy. The synovial fluid was immediately centrifuged at 2,000 g for 10 minutes to remove cells and debris, and the supernatants were aliquoted and rapidly frozen at −80°C until analysis.

### Coomassie blue staining

Equal amount of synovial fluid samples from OA patients and normal controls were diluted by 10 times (1:10 dilution) with lysis buffer containing protease inhibitor (Roche, Basel, Switzerland) and electrophoresed in sodium dodecyl sulfate-polyacrylamide gel electrophoresis (SDS-PAGE; 10% polyacrylamide). The gel was prefixed in 50% MeOH, 10% HoAC, 40% H2O for 30 minutes and then stained with 0.25% Coomassie Brilliant blue R-250 (Bio-Red Laboratories, Hercules, CA) in the above solution for 4 hours. The gel was detained in 5% MeOH, 7.5% HoAC, 87.5% H2O until background was clear. The detained gel was stored in 7% HoAc and a photograph was taken using Canon camera (SD-1000, Canon Inc, Japan).

### Western blotting

After 1:10 dilution, equal amount of synovial fluid samples from OA patients and normal controls were separated using SDS-PAGE under reducing conditions, as previously reported [11]. Once protein bands were transferred to PMSF membrane, The membrane was probed with an antibody against Ihh (1:1,000 dilution) (Abcam, Cambridge, MA), IL-1b (1:1,000 dilution) (R&D systems, Minneapolis, MN), TNF-α (1:1,000 dilution) (Invitrogen, Carlsbad, CA), Fibronectin (1:800 dilution) (Santa Cruz Biotechnology, Santa Cruz, CA), and MMP-13 (1:1,000 dilution) (Abcam, Cambridge, MA). Horseradish peroxidase–conjugated secondary antibody IgG (Bio-Rad) was diluted 1:3,000. Enhanced chemiluminescence (Amersham) was used to visualize immunoreactive proteins. A1AT contents on the same membrane were validated and compared after the membrane was striped and blotted again with A1AT antibody (Abcam, Cambridge, MA).

### Measurement of synovial A1AT

OA synovial fluid (N=10) and normal control synovial fluid (N=11) was diluted by 5000 times (1:5000 dilution), and analyzed by human A1AT ELISA (enzymelinked immunosorbent assay) kit (Bethyl Laboratories, Montgomery, TX). Samples were prepared in triplicates, and test was repeated 3 times.

### Statistical analyses

All data are analyzed using SPSS 13.0 software (SPSS Inc, Chicago, IL). Student’s T test was used for paired group comparisons. P values less than 0.05 were considered statistically significant.

## Results

### Identification of A1AT by mass spectrometry

**Figure 1:**
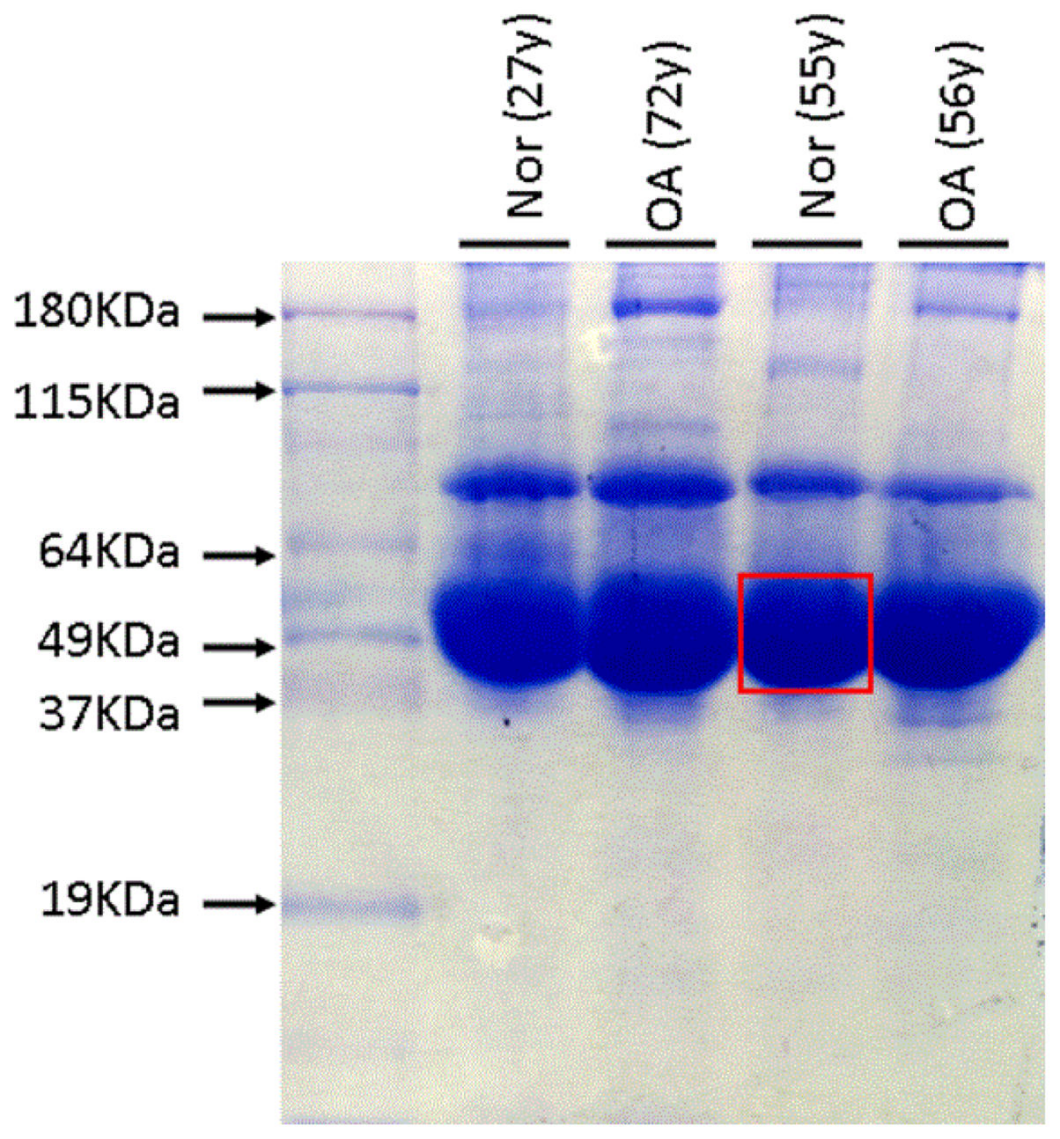
SDS-PAGE gel: Thickest band contains A1AT. Gel fragment inside the white frame was cut down and sent for sequencing. Over 27 sequences compatible to A1AT were identified, suggesting the thickest band on this gel contains A1AT. Normally, 2 repeats are confident enough to confirm identify.

Human synovial fluid samples obtained from OA patients and control group were diluted and separated on a SDS-PAGE gel as described above. On the SDS-PAGE gel, the thickest band was located between 75 kDa and 50 kDa using Coomassie Blue staining and was consistently present in each synovial fluid sample among OA patients and normal controls, suggesting a highly abundant protein (Figure 1). Sequencing result showed that 27 of unique peptide matched A1AT (46 kDa) by Mass Spectrometry, whereas Mass Spectrometry Facility considers any protein with two or more peptide matches confidently identified from our sample. This result provides direct evidence that A1AT is highly abundant in human synovial fluid. In addition, the thickness of A1AT bands in different samples is very close, indicating that A1AT levels are similar among all samples.

### A1AT validation by western blot and ELISA

**Figure 2:**
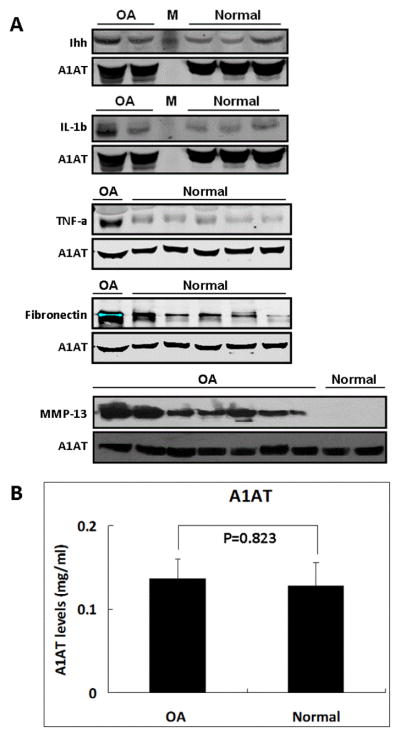
No significant difference of A1AT between OA and normal synovial fluid. (A) Western blot showed constant A1AT levels in OA and normal synovial fluid samples with different contents of Ihh, IL-1β, TNF-α, Fibronectin, and MMP-13. M stands for molecular weight marker. (B) Synovial fluid samples were collected from OA patients (n=10) and normal group (n=11). After 1:5000 dilutions, A1AT content was determined by ELISA. There is no significant difference of A1AT levels between OA and normal synovial fluid samples (p=0.823).

As a good internal control for western blot, A1AT levels should be constant in OA and normal synovial fluids. To test this possibility, western blot and ELISA were performed to determine A1AT levels in OA and normal samples. The differences of Ihh, IL-1b, TNF-α, Fibronectin, and MMP-13 contents between OA and normal synovial fluid samples were detected by western blot while A1AT levels detected on the same membrane remained constant in all samples (Figure 2A). In addition, ELISA did not show significant difference in A1AT contents between synovial fluid from OA patients or normal controls (0.137 ± 0.023 mg/ml and 0.128 ± 0.028 mg/ml, respectively) (Figure 2B).

## Discussion

Previous study has demonstrated that A1AT, a serine protease inhibitor synthesized in the liver, is highly abundant in both blood serum and synovial fluid. Using the quantitative radial immunodiffusion method, Swedlund et al found that A1AT contents in normal and OA synovial fluid are very close [9,12]. In this study, we showed that A1AT protein is constantly present in synovial fluid. Western blot results suggest that A1AT contents in synovial fluid from OA and normal groups are very close even though the contents of Ihh, IL-1b, TNF-α, Fibronectin and MMP-13 are significantly increased in OA synovial fluid samples compared to the normal controls (Figure 2A). Although minor variation in A1AT contents in synovial fluid from patients to patients can be detected by ELISA, there is no significant difference in these samples (Figure 2B). Our result is consistent with previous finding in which there was no significant difference of A1AT level between OA synovial fluid and normal synovial fluid [9].

There is not a well-established loading control for western blot with synovial fluid samples. Many studies present western results without loading control [13–16], while some group use actin as loading control [1]. In general, actin makes a good loading control. However, in synovial fluid, actin levels are not constant. In some RA patients, actin is not present in synovial fluid at all [17]. Hence, it is not appropriate to use actin as a loading control in synovial fluid studies. In contrast, A1AT, which remains largely constant in OA and normal synovial fluid, might be a good candidate.

Although A1AT level is increased in response to inflammatory conditions, such as rheumatoid arthritis [18], there is no need to worry about increased A1AT content in OA synovial fluid. Unlike RA, OA is a non-inflammatory type of arthritis. Although OA is often associated with signs and symptoms of inflammation, including joint pain, swelling, and stiffness, synovial inflammation in OA is mainly limited to small areas adjacent to damaged cartilage and bone [19]. In addition, neutrophils found in RA synovial fluid is not present in OA synovial fluid [19], which implies that increased A1AT level in RA synovial fluid is not necessary in OA. In fact, previous studies and this study both find that A1AT level is relatively constant in OA and normal synovial fluid [9], which is consistent with the fact that OA does not involve severe inflammation.

In summary, our finding is the first study to show that A1AT can be used as a loading control in western blot for human synovial fluid studies. With a good loading control, western results with synovial fluid samples will be more convincing.
